# The Lifestyle Intervention in memory clinics of General and academic Hospitals Trial (LIGHT): Rationale and design of a randomized controlled trial to reduce dementia risk

**DOI:** 10.1002/alz70860_101003

**Published:** 2025-12-23

**Authors:** Veerle van Gils, Sophie C.P.M. Wimmers, Marissa D. Zwan, Lisa Waterink, Judith G.M. Jelsma, Karl G.H. Parisius, Marjolein de Vugt, Robbert J.J. Gobbens, Ron Handels, Sietske A.M Sikkes, Wiesje M. van der Flier, Kay Deckers, Sebastian Köhler, Niels Janssen

**Affiliations:** ^1^ Alzheimer Center Limburg, School for Mental Health and Neuroscience, Maastricht University, Maastricht, Limburg, Netherlands; ^2^ Department of Psychiatry and Neuropsychology, Alzheimer Centrum Limburg, Mental Health and Neuroscience Research Institute (MHeNs), Maastricht University, Maastricht, Netherlands; ^3^ Alzheimer Center Amsterdam, Neurology, Vrije Universiteit Amsterdam, Amsterdam UMC location VUmc, Amsterdam, Netherlands; ^4^ Amsterdam Neuroscience, Neurodegeneration, Amsterdam, Netherlands; ^5^ Amsterdam UMC, location Vrije Universiteit Amsterdam, Department of Public and Occupational Health, Amsterdam, Netherlands; ^6^ Faculty of Health, Sports and Social Work, Inholland University of Applied Sciences, Amsterdam, Netherlands; ^7^ Alzheimer Center Limburg, Mental Health and Neuroscience Research Institute, Maastricht University, Maastricht, Netherlands; ^8^ Zonnehuisgroep Amstelland, Amstelveen, Netherlands; ^9^ Tranzo, Tilburg University, Tilburg, Netherlands; ^10^ Faculty of Behavioural and Movement Sciences, Department of Clinical, Neuro and Developmental Psychology, Vrije Universiteit Amsterdam, Amsterdam, Netherlands; ^11^ Alzheimer Center, Department of Neurology, Amsterdam UMC, Vrije Universiteit Amsterdam, Amsterdam Neuroscience, Amsterdam, Netherlands; ^12^ Department of Epidemiology and Data Science, Amsterdam UMC, Amsterdam, Netherlands

## Abstract

**Background:**

Dementia risk reduction through lifestyle modification has much potential but is not widely implemented in routine care. Patients referred to memory clinics are at high risk for dementia and tend to have worse health and lifestyle profiles. There is currently no service available to help these persons make lifestyle changes. We aim to improve lifestyle profiles through implementing a personalized and multidomain lifestyle intervention in the memory clinic. Here, we describe the design of our study assessing the effectiveness of this intervention.

**Method:**

Lifestyle Intervention in the memory clinics of General and academic Hospitals Trial (LIGHT) is a 12‐month multicentre randomized controlled trial investigating the effects of an innovative lifestyle intervention. Starting early 2025, the trial aims to include 300 older adults (≥ 50 years) with subjective cognitive decline (SCD) or mild cognitive impairment (MCI) who have ≥2 modifiable dementia risk factors (to ensure room for improvement) primarily from the memory clinics of Maastricht UMC+ and Amsterdam UMC. Participants are randomized 1:1 to the intervention or usual care with general health advice. The intervention comprises three components: 1) Three individual sessions with a certified lifestyle coach to work on personal goals, 2) Vouchers for access to brain‐healthy activities from local providers, and 3) Online access to a recently developed risk self‐management platform (www.breinzorg.nl). Primary outcome is the 12‐month change in participants dementia risk profiles as measured by the LIfestyle for BRAin Health (LIBRA) index. Secondary outcomes include cognitive functioning, health‐related quality of life, activities of daily living, psychological measures, as well as individual lifestyle components.

**Result:**

LIGHT provides insight into the implementability and (cost‐) effectiveness of a lifestyle intervention in a memory clinic setting. Recruitment has started early 2025 and will continue until 2027.

**Conclusion:**

LIGHT may provide evidence for a personalized multidomain lifestyle intervention for preventing dementia. The results would support implementing personalized lifestyle coaching in the regular service offered by Dutch memory clinics.

